# Effects of Different Concentrations of Oil Mist Particulate Matter on Pulmonary Fibrosis In Vivo and In Vitro

**DOI:** 10.3390/toxics10110647

**Published:** 2022-10-28

**Authors:** Huipeng Nie, Huanliang Liu, Yue Shi, Wenqing Lai, Xuan Liu, Zhuge Xi, Bencheng Lin

**Affiliations:** Tianjin Institute of Environmental and Operational Medicine, Tianjin 300050, China

**Keywords:** OMPM, TGF-β1/Smad3, TGF-β1/MAPK p38, PF

## Abstract

**Highlights:**

**What are the main findings?**

- OMPM exposure induced lung lesions and PF in rats.

**What is the implication of the main finding?**

- OMPM exposure induced PF via TGF-β1/Smad3 and TGF-β1/p38 MAPK signaling pathways.
- OMPM exposure induced fibrotic phenotypes in the BEAS-2B cell line.
- OMPM exposure activated the differentiation of the HFL-1 cell line.
- OMPM exposure led to the BEAS-2B cell line being able to induce the differentiation of the HFL-1 cell line.

**Abstract:**

Oil-mist particulate matter (OMPM) refers to oily particles with a small aerodynamic equivalent diameter in ambient air. Since the pathogenesis of pulmonary fibrosis (PF) has not been fully elucidated, this study aims to explore the potential molecular mechanisms of the adverse effects of exposure to OMPM at different concentrations in vivo and in vitro on PF. In this study, rats and cell lines were treated with different concentrations of OMPM in vivo and in vitro. Sirius Red staining analysis shows that OMPM exposure could cause pulmonary lesions and fibrosis symptoms. The expression of TGF-β1, α-SMA, and collagen I was increased in the lung tissue of rats. The activities of MMP2 and TIMP1 were unbalanced, and increased N-Cadherin and decreased E-Cadherin upon OMPM exposure in a dose-dependent manner. In addition, OMPM exposure could activate the TGF-β1/Smad3 and TGF-β1/MAPK p38 signaling pathways, and the differentiation of human lung fibroblast HFL-1 cells. Therefore, OMPM exposure could induce PF by targeting the lung epithelium and fibroblasts, and activating the TGF-β1/Smad3 and TGF-β1/MAPK p38 signaling pathways.

## 1. Introduction

Pulmonary fibrosis (PF) is a complex pathophysiological process that is common to most interstitial lung diseases. The pathological characteristics of pulmonary fibrosis are the diffuse chronic inflammation of alveolar walls and interstitial fibrosis; eventually, the pulmonary parenchyma is gradually replaced by fibrous scar tissue and the loss of respiratory function [1,2]. As the disease progresses, patients with pulmonary fibrosis eventually die due to respiratory failure. In recent years, the incidence of pulmonary fibrosis has increased significantly in China and abroad, and the incidence of pulmonary fibrosis in China is 3–5/100,000 per year [3,4]. The early diagnosis of pulmonary fibrosis is difficult due to its insidious onset and no specific clinical manifestations [5]. In addition, there is no special treatment for PF, resulting in high mortality rates of patients with PF. Epidemiological studies confirmed that environmental factors may induce PF, and participate in the occurrence and development of pulmonary interstitial fibrosis [6,7]. For example, the exposure to metal dust and wood dust can lead to increased risk of pulmonary interstitial fibrosis [8,9].

Oil-mist particulate matter (OMPM) refers to oily particles with a small aerodynamic equivalent diameter in ambient air, which is one of the main pollution factors in the urban atmosphere [10]. Due to the small particles, OMPM can enter the deep respiratory tract and deposit in all levels of the bronchus and alveoli [11]. At the same time, OMPM have a larger surface-area-to-volume ratio, which can adsorb more pathogenic microorganisms, acid oxides, transition metal components, and polycyclic aromatic hydrocarbons, resulting in increased biological toxicity [4,12]. A large number of studies confirmed that exposure to particulate matter in ambient air is closely related to the increased morbidity and mortality of respiratory diseases [13]. OMPM exposure can be involved in the occurrence and development of pulmonary infection, COPD, and other respiratory diseases through pulmonary inflammation and oxidative stress [13,14]. However, it is not clear whether oil-mist exposure is involved in the occurrence and development of PF [15]. In recent decades, with the development of the global industry and the process of globalization, marine resources have become the commanding heights of human exploitation and utilization. With the increase in large-scale operations conducted by humans at sea, the OMPM pollution emitted from these operations is becoming increasingly serious. As a result, the local marine environment has undergone great changes and continues to expand. At the same time, the entry of OMPM into the air and marine environment also poses a serious threat to marine life and human health. Furthermore, China’s regulations for offshore operations are as follows: the instantaneous discharge of washing water for offshore operations must not exceed 30 L/kN, and the total discharge must not exceed 1/30,000th of the cargo capacity of the last voyage [11,16]. A large amount of oil pollution discharge adversely affects the marine ecological environment and the health of offshore workers [17]. In recent years, the relationship between OMPM and ecological health has become a hot global issue.

The pathological basis of early pulmonary fibrosis is acute alveolitis, which involves inflammatory cells, immune effector cells, and cytokines in the formation of alveolitis [7,18]. Recent studies found that a variety of cytokines play an important role in the formation of fibrosis [2,5]. These cytokines include promoting and inhibiting fibrosis. They coordinate and resist each other, and determine the outcome of lung-tissue injury and repair. Pulmonary fibrosis is a disease in which the functions of the two major types of factors lose balance during the repair of a lung injury, resulting in the damage of lung function and tissue structure [19]. TGF-β1 is a key cytokine causing fibrosis that can promote excessive proliferation and differentiation of lung fibroblasts, and then promote the excessive accumulation of extracellular matrices such as collagen in the interstitium and lung alveoli, leading to the occurrence and development of pulmonary fibrosis [20]. Studies showed that increased TGF-β1 levels are found in multifactorial pulmonary fibrosis, including bleomycin, cyclophosphamide, and radiation [2,21]. TGF-β1 content was closely correlated with pulmonary fibrosis. Numerous studies found that TGF-β1 can induce the transformation of fibroblasts into myofibroblasts by promoting the expression of Smad3 and p38MAPK, can stimulate the mitosis and synthesis and deposition of pulmonary fibroblasts, and induce the occurrence of pulmonary fibrosis [22,23]. Therefore, the TGF-β1/Smad3 and TGF-β1/p38 MAPK signaling pathways are the most important pathways promoting the formation of PF [24].

In our study, rats and cell lines were treated with different concentrations of OMPM to establish PF models in vivo and in vitro, and the molecular mechanism of TGF-β1/Smad3 and TGF-β1/p38 MAPK signaling pathways of the occurrence and development of PF induced by OMPM exposure was studied indepth using histopathology, molecular biology, and cell biology.

## 2. Materials and Methods

### 2.1. OMPM Collection

This study was carried out in a large offshore operation. A large amount of OMPM was collected during the working hours of the offshore operation. As described in previous studies (Nie et al., 2022 [25]), a media flow sampler (100 L/min) and a quartz filter (Whatman, UK, φ90 mm) were used to collect the OMPM. Before sampling, a blank quartz-filter membrane was heated in a muffle furnace at 550 °C for 4 h to remove the residual organic matter and other organic impurities. After balancing in a dryer for 24 h, we weighed the film on 1/10,000 scales. After sampling, the filter membrane was placed in a dryer for 24 h and weighed.

### 2.2. Animal Model and Grouping

SPF Wistar rats (6–8 weeks old, 200 ± 20 g) were purchased from Beijing Vital River Laboratory Animal Technology Co., Ltd. (Beijing, China) The animals were fed in the Experimental Animal Center of Tianjin Institute of Environmental and Occupational Medicine. We followed the regulations of the Experimental Animal Ethics Committee of the Tianjin Institute of Environmental and Occupational Medicine (IACUC of AMMS-04-2020-016). The rats drank water and ate freely. The breeding condition of the rats was room temperature at 24 ± 2 °C and humidity at 50% ± 10%.

To establish the animal model, the rats were directly exposed to different concentrations of OMPM. According to the dose per unit body weight, the equivalent dose of OMPM in rats was equivalent to 6.3 times that of humans. Therefore, the dose of OMPM in rats was 16 mg/m^3^ multiplied by 6.3, which was about 100 mg/m^3^. After about a week’s acclimation, the rats were randomly divided into 3 groups: the high concentration of OMPM (HC group, the concentration of OMPM was 100 mg/m^3^), low concentration of OMPM (LC group, the concentration of OMPM was 50 mg/m^3^ for half treatment of high concentration) and control (the concentration of OMPM was 0 mg/m^3^) groups, with 18 rats from each sex in each group, and OMPM exposure was 6.5 h a day for 42 consecutive days.

### 2.3. Cell Culture and Treatment

Human lung epithelial cell line BEAS-2B and human lung fibroblast cell line HFL-1 were purchased from the Institute of Biochemistry and Cell Biology, Chinese Academy of Sciences (Shanghai, China). The cell lines were cultured in a DMEM medium (Solarbio, Beijing, China) supplemented with 10% fetal bovine serum (FBS, Gibco, China) in a 37 °C incubator. A single-concentration cell exposure system (HRH-CES1332, Beijing Huironghe Technology Co., Ltd., Beijing, China) was used to expose the cells of BEAS-2B and HFL1 to OMPM for 1 h at different concentrations of 0, 5, 10, 20, 30, 40 mg/m^3^.

### 2.4. Histopathology

The rat lung tissues were fixed in 4% paraformaldehyde (PFA) for 24 h, and the tissue paraffin slides were prepared. The paraffinized lung slides were deparaffinized and stained with Sirius Red to observe the tissue morphology. The images were obtained using a bioelectron microscope (OLYMPUS, Tokyo, Japan, BX51).

### 2.5. Immunofluorescence

The different groups of BEAS-2B and HFL-1 cell lines were used to prepare the paraffin slides. After dewaxing and dehydrating, antigen retrieval was performed at 95 °C for 10 min. The paraffin slices were blocked with 5% BSA at room temperature for 1 h. For immunofluorescence, FITC-labeled fluorescent primary antibody collagen I (Affinity, Jiangsu, China, dilution: 1:200) and α-SMA (CST, Boston, USA, dilution: 1:200) were incubated overnight at 4 °C. On the next day, sections were rewarmed, washed with PBS, and antirabbit IgG (H + L) Alexa Fluor^®^ 488 conjugate secondary antibody (CST, Boston, USA, dilution: 1:500) was incubated in the dark for 1 h. After restaining with DAPI, the slices were observed with a fluorescence microscope (OLYMPUS, Japan, BX51) and photographed. The percentage of fluorescently labeled cells in rat lung tissues was counted with different fluorescently labeled cell-counting methods and the fluorescence intensity statistics of Image J. In addition, we anonymously shuffled the image order statistics to prevent statistical preference. Three fields of view were also analyzed. The magnification was 100×.

### 2.6. Western Blotting

The rat lung tissues and cell samples were scraped and resuspended in a 0.5 mL RIPA buffer (CWBIO, Jiangsu, China). Tissue or cell lysates were placed on ice for 1 h. After centrifugation at 10,000× *g* for 20 min, the protein concentration was determined using the BCA kit (Beyotime, Shanghai, China). Then, 20 μg samples were electrophoresed via 10% SDS-PAGE (Yeasen Biotechnology, Shanghai, China) and transferred onto a PVDF membrane. Samples were blocked with 5% nonfat milk in TBST solution at room temperature for 2 h and then probed with primary antibodies p38 (CST, Boston, USA, dilution: 1:1000), p-p38 (CST, Boston, USA, dilution: 1:1000), Smad3 (CST, USA, dilution: 1:1000), p-Smad3 (CST, Boston, USA, dilution: 1:1000), GAPDH (Utibody, Tianjin, China, dilution: 1:2000) at 4 °C overnight. Secondary antibodies used for detection included HRP-conjugated antirabbit IgG and antimouse IgG (Bioss, Beijing, China, dilution: 1:3000), and the protein expression was detected using an ECL chemiluminescence detection kit (Boster, Wuhan, China).

### 2.7. ELISA Assay

The rat serum or lung tissues and cell culture medium supernatants were obtained, and the samples were stored at −80 °C. α-SMA, COL1, N-cadherin, E-cadherin, MMP-2, and TIMP1 activities were determined using ELISA kits (Cloud-Clone, Beijing, China).

### 2.8. Statistical Analysis

Classical scientific software SPSS 25.0 was used for statistical data analysis. The data are expressed as mean ± standard deviation. The t-test was used for comparison between the two groups, and one-way ANOVA was used among the multiple groups. and comparisons between groups were performed using LSD (homogeneity of variance) or Dunnett’s T3 (non-homogeneity of variance). *p* < 0.05 was considered to be statistically significant. The analyzed data were plotted using GraphPad Prism 5.0 software.

## 3. Results and Discussion

### 3.1. OMPM Exposure Induced Lung Lesions and PF in Rats

**Figure 1:**
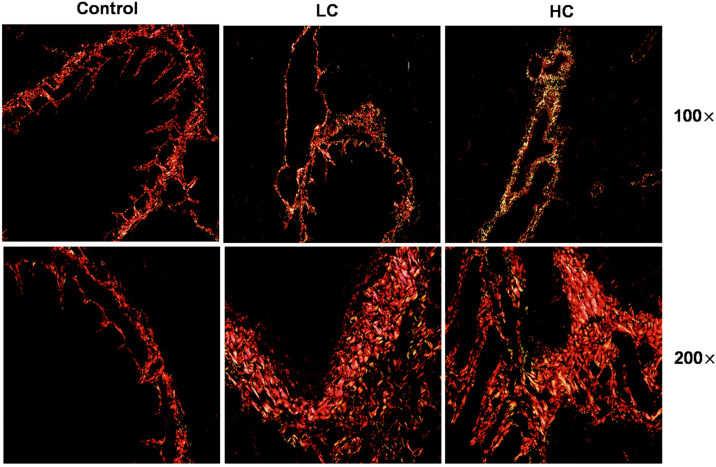
OMPM-induced PF in Wistar rat lung tissues with Sirius Red staining; 100×, bar: 100 µm; 200×, Bar: 50 µm.

To investigate the potential effect of OMPM exposure on PF induction, the rats exposed to OMPM were examined histologically. According to our previous study (Nie et al., 2022 [25]), the OMPM was composed of tiny particles with various shapes and structures, with very irregular shapes, an uneven surface, uneven distribution, and different particle sizes. Otherwise, OMPM came in granular, blocky, strip, column, needle, and lamellar shapes, most of which clustered together, and some particles even overlapped. In addition, according to previous EDS data analysis (Nie et al., 2022 [25]), OMPM was mainly composed of aluminosilicate, inorganic salt crystals, organic carbon, random carbon, and other substances. The structure of a substance determines the properties; therefore, we found via HE staining that the long-term exposure to OMPM might trigger PF (Nie et al., 2022 [25]). Sirius Red staining is a classical pathological method to observe the distribution of collagen in tissues. Compared with the control group, the expression of collagens I and III in the lung tissues of rats in the HC and LC groups was significantly upregulated (Figure 1), indicating that the lung tissues of the rats in these two groups presented interstitial lung lesions and pulmonary inflammation with PF-like symptoms. In addition, in the HC group, the degree of lung-tissue lesions and fibrosis was more serious, which may have been due to the long-term exposure of high concentrations of OMPM to aggravate the lung injury of rats. There are few pathological studies on the effect of PM2.5 exposure on pulmonary fibrosis in animals. Hirofumi Kamata et al. confirmed that 14 nm carbon black nanoparticles could aggravate lung inflammation and fibrosis in rats, which is consistent with our results [26,27].

**Figure 2:**
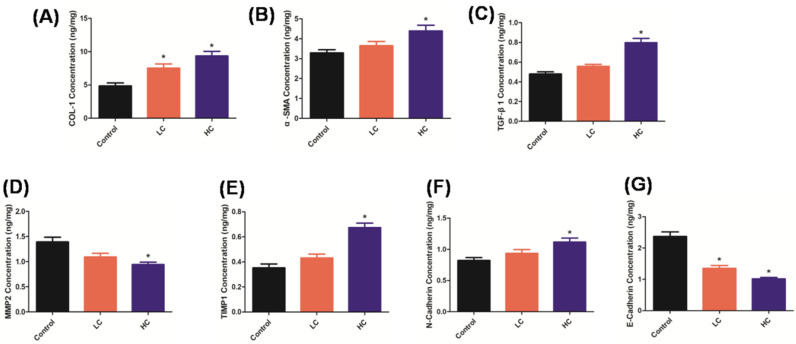
PF biomarkers were determined via ELISA assay after OMPM exposure at different concentrations in rat serum and lung tissues. (**A**) COL-1 concentration; (**B**) α-SMA concentration; (**C**) TGF-β1 concentration; (**D**) MMP2 concentration; (**E**) TIMP1 concentration; (**F**) N-cadherin concentration; (**G**) E-cadherin concentration. Data represent mean ± SD. * *p* < 0.05 vs. the control group.

Furthermore, Figure 2A–C show the classical PF biomarkers via an ELISA assay. We observed significant accumulation of COL1, α-SMA, and TGF-β1 in rat lung tissues after OMPM treatment, suggesting that OMPM may induce the extensive activation of lung fibroblasts and the accumulation of an extracellular matrix, thereby triggering the occurrence of PF. Meanwhile, compared with the LC group, the PF lesions were more serious in the HC group, which is consistent with the Sirius Red staining results, and also indicates that OMPM induced PF in a dose-dependent manner. In addition, Matrix metalloproteinase (MMP) and the tissue inhibitor of metalloproteinase (TIMP) are two important factors in the synthesis and degradation regulation of the extracellular matrix (ECM) [28]. MMP-2 mainly degrades collagens I and Ⅲ, the main components of the extracellular matrix, and TIMP-1 can inhibit the activity of MMP-2 [29]. In the process of pulmonary fibrosis, MMP-2 activity decreases, while TIMP-1 activity increases continuously. Previous studies showed that the imbalance of the two activities is an important factor leading to the progression of PF [22]. In our study, compared with the control group, a significant imbalance of MMP-2 and TIMP-1 activity occurred (Figure 2D,E), indicating the occurrence of PF upon OMPM exposure. Moreover, with increased N-cadherin and decreased E-cadherin compared to the control rats (Figure 2F,G), two EMT and PF biomarkers further validated the OMPM-induced lung lesions and PF in rats. Taken together, these results indicate that OMPM might cause significant lung lesions and PF symptoms in a dose-dependent manner in vivo. In the rat model of lung injury and fibrosis constructed in this study, TGF-β1 was significantly increased in the peripheral blood, which was consistent with the changes in COL1 and α-SMA. TGF-β1 could promote intercellular adhesion and cross-cell interaction, and amplify the inflammatory cascade. Previous studies showed that TGF-β1 knockdown attenuates bleomycin-induced inflammatory cell infiltration and pulmonary fibrosis in rats, and attenuates fibrosis-related mediators [30,31].

### 3.2. OMPM Exposure Induced PF via the TGF-β1/Smad3 and TGF-β1/p38 MAPK Signaling Pathways

**Figure 3:**
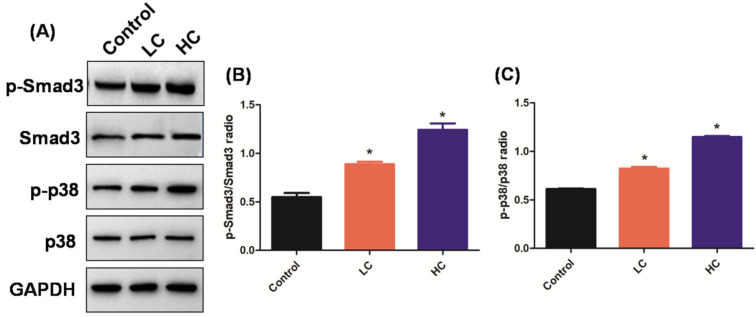
**OMPM exposure induced PF via the TGF-β1/Smad3 and TGF-β1/p38 MAPK signalingpathwaysin rat lung tissues.** (**A**) The phosphorylation of Smad3 and p38 after OMPM exposure detected with Western blotting; (**B**) ratio of p-Smad3/Smad3; (**C**) ratio of p-p38/p38. Data represent mean ± SD. * *p* < 0.05 vs. the control group.

TGF-β1 is mainly composed of eosinophils, macrophages, fibroblasts, and secreted muscle fibroblasts. The TGF-β1/Smad3 and TGF-β1/p38 MAPK signaling pathways are keys to normal wound repair and repair-regulating mechanisms [15,32]. The signaling pathways are characteristic of many fibrosis diseases, and are fibroblast recruiters, activating and differentiating the central adjustment, thus playing a core role in the pathogenesis of PF [14,33]. A previous study found that the upregulation of the TGF-β1/Smad3 signaling pathway in the PM2.5 exposure group was significantly higher than that in the control group at each time point, suggesting that PM2.5 exposure aggravated PF by increasing the TGF-β1 [23,29]. However, it is not clear whether OMPM could induce PF via TGF-β1/Smad3 and TGF-β1/p38 MAPK signaling pathways. To solve this mystery, TGF-β1/Smad3 and TGF-β1/p38 MAPK signaling pathways were observed with Western blotting. Consistent with what we suspected, compared with the control group, there was a significant increase in the p-Smad3/Smad3 and p-p38/p38 ratios in the HC and LC groups (Figure 3A), which indicated that the TGF-β1/Smad3 and TGF-β1/p38 MAPK signaling pathways were activated, and the degree of activation of the TGF-β1/Smad3 and TGF-β1/p38 MAPK signaling pathways in the HC group was higher than that in the LC group (Figure 3B,C), which is consistent with the previous experimental data. Therefore, OMPM exposure may have induced PF via the TGF-β1/Smad3 and TGF-β1/p38 MAPK signaling pathways. In recent years, with the deepening of research on the structure and biological function of Smad3 and p38 MAPK, the role of Smad3 and p38 MAPK in organ fibrosis has gradually attracted attention [34]. Studies confirmed that the formation of the Smad3 heterodimer and the nuclear translocation of p38 MAPK are key links that are the biomarkers of the activation of the TGF-β1/Smad3 and TGF-β1/p38 MAPK signaling pathways [35]. Therefore, TGF-β1/Smad3 and TGF-β1/p38 MAPK signaling pathways play an important role in the PF and are the most closely related to the process of PF.

### 3.3. OMPM Exposure Induced Fibrotic Phenotypes in the BEAS-2B Cell Line

**Figure 4:**
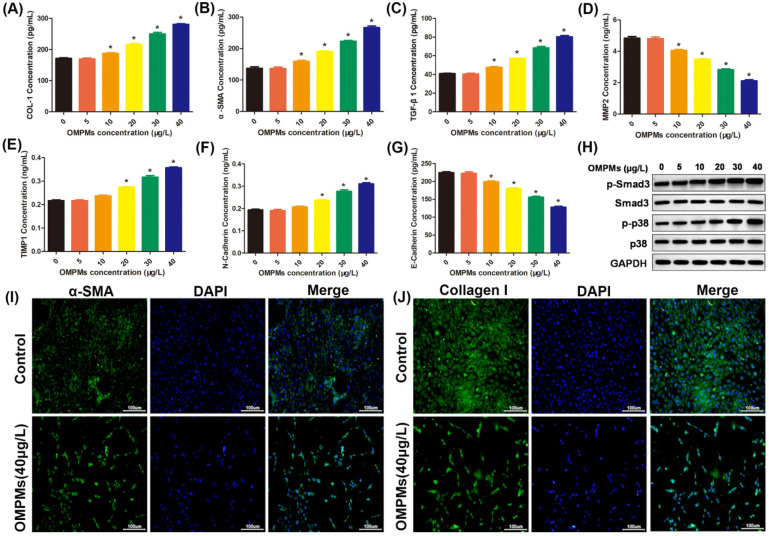
**OMPMexposure induced fibrotic phenotypes in the BEAS-2B cell line.** (**A**) COL-1 concentration; (**B**) α-SMA concentration; (**C**) TGF-β1 concentration; (**D**) MMP2 concentration; (**E**) TIMP1 concentration; (**F**) N-cadherin concentration; (**G**) E-cadherin concentration. (**H**) Phosphorylation of Smad3 and p38 after OMPM exposure detected with Western blotting. (**I**) α-SMA expression after OMPM exposure was detected using immunofluorescence. (**J**) Collagen I expression after OMPM exposure was detected using immunofluorescence; 100×, bar: 100 µm. Data represent mean ± SD. * *p* < 0.05 vs. the control group.

Inhalable particulate matter could come into direct contact with and injure the bronchi after being inhaled into the human body, resulting in the inflammation and lesions of lung epithelial tissue, which is one of the main causes of PF [17,19]. Therefore, in the in vitro experiments, human bronchial epithelial BEAS-2B cell line was used to study the relationship between an OMPM-exposed lung epithelial cell line and fibrotic phenotypes. After the exposure of BEAS-2B cells to different types of OMPM, compared with the control group, ELISA and imunofluorescence showed that α-SMA, collagen I, and TGF-β1 levels in OMPM-treated cells were significantly increased and dose-dependent (Figure 4A–C,I,J), indicating that the high concentration of OMPM was more direct to damage to the BEAS-2B cell line. We further measured the levels of MMP2, TIMP1, and E-cadherin in the BEAS-2B cell line with ELISA. Alveolar macrophages can release some cytokines and proinflammatory factors under the imbalance of MMP2 and TIMP1, which can, in turn, further stimulate fibroblast and lung epithelial cells to secrete a variety of cytokines, such as IL-8, IL-6, and adhesion factors. At the same time, the imbalance of MMP2 and TIMP1 can cause varying inflammatory cell aggregation, and may have a profound effect on the pulmonary inflammatory response and fibrous hyperplasia. As shown in Figure 4D–G, in OMPM-exposed cells, the level of MMP2 was largely inhibited, and the level of TIMP1 was largely improved, suggesting that the imbalance of MMP2 and TIMP1 activities might induce the occurrence of PF upon OMPM exposure. Moreover, in previous studies, N-cadherin significantly increased in the peripheral blood, consistent with TGF-β1, and procollagens I and III, which promoted intercellular adhesion and intercellular interaction, and amplifying the inflammatory cascade. In our study, increased N-cadherin and decreased E-cadherin further validated that OMPM exposure could induce PF in the BEAS-2B cell line. Additionally, there was a significant increase in p-Smad3/Smad3 and p-p38/p38 ratios upon OMPM exposure in the BEAS-2B cell line, with a dose-dependent tendency (Figure 4H). Taken together, these data suggest that OMPM exposure could induce fibrotic phenotypes in the BEAS-2B cell line, which is in perfect agreement with the results of the animal experiments.

### 3.4. OMPM Exposure Activated the Differentiation of the HFL-1 Cell Line

**Figure 5:**
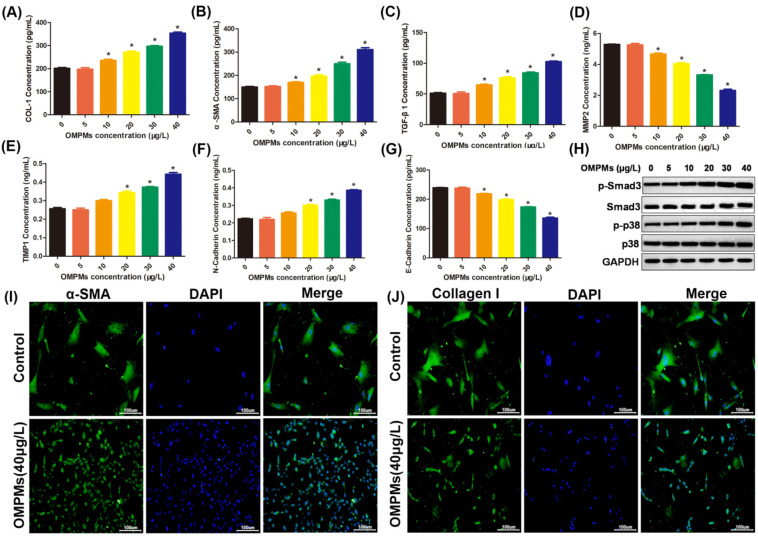
OMPM exposure activated the differentiation of the HFL-1 cell line. (**A**) COL-1 concentration; (**B**) α-SMA concentration; (**C**) TGF-β1 concentration; (**D**) MMP2 concentration; (**E**) TIMP1 concentration; (**F**) N-Cadherin concentration; (**G**) E-cadherin concentration. (**H**) Phosphorylation of Smad3 and p38 after OMPM exposure detected with Western blotting. (**I**) α-SMA expression after OMPM exposure was detected using immunofluorescence. (**J**) Collagen I expression after OMPM exposure was detected using immunofluorescence; 100×, bar: 100 µm. Data represent mean ± SD. * *p* < 0.05 vs. the control group.

The massive differentiation of lung fibroblasts is the main cause of lung-tissue inflammation and pulmonary fibrosis [1,36]. Therefore, we chose the HFL-1 cell line as the research object to explore whether OMPM could induce the differentiation of lung fibroblasts. As shown in Figure 5A–C, different concentrations of OMPM exposure triggered a dose-dependent increase in the expression of TGF-β1, collagen I, and α-SMA in the HFL-1 cell line. Moreover, the imbalance of MMP2 and TIMP1, and increased N-cadherin and decreased E-cadherin further validated that OMPM exposure could activate the differentiation of the HFL-1 cell line (Figure 5D–G), suggesting that OMPM exposure might exacerbate the ECM deposition and EMT process in the HFL-1 cell line. Furthermore, immunofluorescence assay verified that a treatment with a high concentration of OMPM (40 mg/m^3^) caused a significant increase in the expression of myofibroblast markers α-SMA and collagen I (Figure 5I,J). A significant increase in p-Smad3/Smad3 and p-p38/p38 ratios was observed after OMPM treatment (Figure 5H), which might have contributed to the rapid profibrotic activation to OMPM in the HFL-1 cell line. Taken together, these data indicate that OMPM promoted the activation of the TGF-β1/Smad3 and TGF-β1/p38 MAPK signaling pathways, and lung fibrotic differentiation phenotypes in the HFL-1 cell line.

### 3.5. OMPM Exposed BEAS-2B Cell Line Could Induce Differentiation of HFL-1 Cell Line

**Figure 6:**
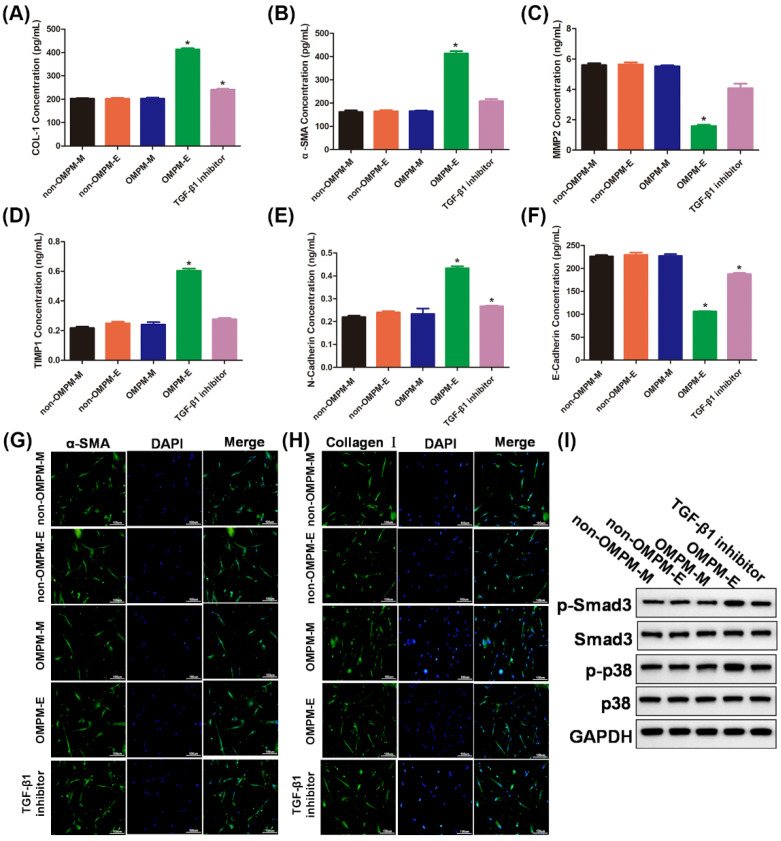
**Different ways with which OMPM-exposed BEAS-2B cell lines could induce the differentiation of the HFL-1 cell line.** (**A**) COL-1 concentration; (**B**) α-SMA concentration; (**C**) MMP2 concentration; (**D**) TIMP1 concentration; (**E**) N-cadherin concentration; (**F**) E-cadherin concentration. (**G**) α-SMA expression after the treatment of the medium of non-OMPM-M and OMPM-M cell supernatants of non-OMPM-E and OMPM-E groups of BEAS-2B cell lines was detected using immunofluorescence. (**H**) Collagen I expression after the treatment of the medium of non-OMPM-M and OMPM-M cell supernatants of non-OMPM-E and OMPM-E groups of BEAS-2B cell lines was detected using immunofluorescence; 100×, bar: 100 µm. (**I**) Phosphorylation of Smad3 and p38 after the treatment of the medium of non-OMPM-M and OMPM-M cell supernatants of non-OMPM-E and OMPM-E groups of BEAS-2B cell lines was detected with Western blotting. Non-OMPM-M, medium for BEAS-2B without OMPM treatment before it was used for culture; OMPM-M, medium for BEAS-2B with OMPM treatment before it was used for culture; non-OMPM-E, BEAS-2B that was not exposed to OMPM; OMPM-M, BEARS-2B that was exposed to OMPM. Data represent mean ± SD. * *p* < 0.05 vs. the non-OMPM-M, OMPM-M, and non-OMPM-E groups.

Dysfunctional lung epithelial cells often cause pulmonary fibrosis by interacting with lung fibroblasts [37]. Therefore, we hypothesized that OMPM-induced pulmonary epithelial dysfunction might also lead to pulmonary fibrosis phenotypes by interacting with pulmonary fibroblasts. To assess this hypothesis, a culture medium of non-OMPM-M and OMPM-M, cell supernatants of non-OMPM-E and OMPM-E groups of BEAS-2B cell lines were used to incubate the HFL-1 cell line. As shown in Figure 6A, B, compared with the non-OMPM-M, OMPM-M, and non-OMPM-E groups, the medium supernatants of the OMPM-E groups of the BEAS-2B cell line triggered an increase in the expression of collagen I and α-SMA in the HFL-1 cell line. Once the TGF-β1 inhibitor had been added, however, the levels of collagen I and α-SMA returned to normal. Similar to previous results, an imbalance of MMP2 and TIMP1, increased N-cadherin, and decreased E-cadherin further validated that the medium supernatants of the OMPM-E groups of the BEAS-2B cell line could activate the differentiation of the HFL-1 cell line, and the TGF-β1 inhibitor had the same effect (Figure 6C–F). Furthermore, immunofluorescence staining showed that, compared with the non-OMPM-M, OMPM-M, non-OMPM-E, and TGF-β1 inhibitor groups, the expression of α-SMA and collagen I was increased in the cell supernatants of the OMPM-E group-treated HFL-1 cell line (Figure 6G,H). Consistent with immunofluorescence staining, Western blotting showed that a significant increase in the p-Smad3/Smad3 and p-p38/p38 ratios was observed after the treatment of the medium supernatants of the OMPM-E group of the BEAS-2B cell line, indicating that the OMPM-exposed BEAS-2B cell line could activate the TGF-β1/Smad3 and TGF-β1/p38 MAPK signaling pathways (Figure 6I). The results confirm our hypothesis that the OMPM-exposed BEAS-2B cell line could promote the activation of the TGF-β1/ Smad3 and TGF-β1/p38 MAPK signaling pathways and lung fibrotic differentiation phenotypes in the HFL-1 cell line. The activation of the TGF-β1/ Smad3 and TGF-β1/p38 MAPK signaling pathways, and lung fibrotic differentiation phenotypes in the HFL-1 cell line can be regarded as the initial event of PF in vitro [38,39].

## 4. Conclusions

Our work systematically demonstrated that OMPM plays an important role in inducing PF in vivo and in vitro. First, OMPM could cause lung tissue lesions and PF in rats. In addition, in the in vitro cell experiments, we further confirmed the molecular mechanism of the profibrotic effect of OMPM, and OMPM exposure could trigger the EMT development and fibroblast differentiation of pulmonary bronchial epithelial cells. Therefore, our work provides a theoretical and practical basis for filling in the blank of whether there is a health risk of OMPM in offshore operations, and has constructive significance for the sustainable development of offshore operations.

## Data Availability

Not applicable.
